# Cholesterols Work as a Molecular Regulator of the Antimicrobial Peptide-Membrane Interactions

**DOI:** 10.3389/fmolb.2021.638988

**Published:** 2021-02-03

**Authors:** Jia Li, Xuemei Lu, Wendong Ma, Zhonglan Chen, Shuqing Sun, Qinghui Wang, Bing Yuan, Kai Yang

**Affiliations:** ^1^ Electric and Informative Engineering School, Yunnan Minzu University, Kunming, China; ^2^ Wenzheng College of Soochow University, Suzhou, China; ^3^ Center for Soft Condensed Matter Physics and Interdisciplinary Research & School of Physical Science and Technology, Soochow University, Suzhou, China

**Keywords:** cholesterol, antimicrobial peptide, membrane poration, molecular dynamics, GUV leakage, cytotoxicity

## Abstract

The existing cholesterols (Chols) in animal cell membranes play key roles in many fundamental cellular processes, which also promise the possibility to modulate the bioactivity of various membrane-active biomacromolecules. Here, combining dynamic giant unilamellar vesicle leakage experiments and molecular dynamics simulations, the inhibitory effect of Chols on the membrane poration activity of melittin (Mel), a typical natural antimicrobial peptide, is demonstrated. Molecular details of the Mel-Chol interactions in membrane show that, for a Chol-contained lipid membrane, Mel exposure would perturb the symmetric bilayer structure of the membrane and specifically influence the location and orientation distributions of Chol molecules to an asymmetric state between the two leaflets; moreover, the Mel-Chol interactions are significantly influenced by the membrane environment such as unsaturation degree of the lipid components. Such inhibitory effect is normally ascribed to an accumulation of Chol molecules around the membrane-bound peptide chains and formation of Chol-Mel complexes in the membrane, which hinder the further insertion of peptides into the membrane. This work clarifies the molecular interactions between membrane-active peptides and Chol-contained membranes, and suggest the possibility to develop targeted drugs due to the membrane component specificity between bacterial and animal cells.

## Introduction

Antibiotic resistance crisis has becoming a world-wide healthcare threat for human beings and therefore the development of new bactericides is urgently needed (Willyard, 2017; Lam et al., 2016). Antimicrobial peptides (AMPs), an effective weapon of the innate immune system to protect the host from bacterial invasion, promise a fundamental resolution to this serious issue (Lu et al., 2019b; Munguia and Nizet, 2017; Sancho-Vaello and Zeth, 2015; Xiao et al., 2019; Zhang et al., 2018). AMPs kill bacteria by directly permeabilizing the bacterial membranes leading to leakage of cellular content (Hong et al., 2019; Lee et al., 2013; Ma et al., 2020; Wimley, 2010). Such a unique action mechanism (i.e., physical damage to cellular membranes) barely induce any drug resistance (Lázár et al., 2018; Lee et al., 2013; Liu et al., 2018; Lu et al., 2012; Sancho-Vaello and Zeth, 2015). However, at present the clinical use of AMPs is blocked due to their potential cytotoxicity to mammalian cells (Sancho-Vaello and Zeth, 2015; Xu et al., 2015). Therefore, molecular understanding of the antibacterial mechanism and realization of targeted attack to the specific bacterial membranes are important for the practical use of them.

As one of the most representative AMPs, the bee venom peptide melittin (Mel) consists of 26 amino acid residues (Gly(+)-Ile-Gly-Ala-Val^5^-Leu-Lys(+)-Val-Leu-Thr^10^-Thr-Gly-Leu-Pro-Ala^15^-Leu-Ile-Ser-Trp-Ile^20^-Lys(+)-Arg(+)-Lys(+)-Arg(+)-Gln^25^-Gln-NH_2_) which forms an amphipathic α-helical structure when binding on a cell membrane (Hong et al., 2019; Wimley, 2010; Yang et al., 2001). The positively-charged amphipathic structure, which is regarded as the most characteristic configuration of AMPs, makes Mel a widely-used peptide model for studying the membrane activity of AMPs (Huang et al., 2004; Wimley, 2010; Liu et al., 2018; Hong et al., 2019; Lu et al., 2019b). Specifically, in recent years, much attention is paid on Mel due to its potent antimicrobial activity and tumor cell killing or even HIV destroying abilities (Duffy et al., 2020; Memariani et al., 2019; Ramadurai et al., 2009). As a result of the complicated Mel-lipid interactions, to form transmembrane pores is a premise of the function realization of Mel (Hong et al., 2019; Lee et al., 2013; Krauson et al., 2012). Such an interaction process generally obeys the two-state model, including the binding and accumulation of peptides on membrane surface and the subsequent transmembrane insertion of peptides for pore formation (Huang, 2000; Lee et al., 2013; Xu et al., 2020). This process is influenced by many factors especially the peptide concentration, i.e., the peptide-to-lipid ratio, P/L (Hong et al., 2019). At a low P/L ratio, peptides solely adsorb on the membrane surface. Once the P/L ratio is above a threshold value (e.g., P/L = 1/43), some peptides accumulate together and corporately reorient themselves to achieve membrane insertion which finally cause pore formation (Lee et al., 2013). Furthermore, transition of peptides from the membrane surface-binding state to transmembrane-inserting state is accompanied with the changes in lipid packing states (e.g., membrane thinning and stretching) (Hong et al., 2019; Lee et al., 2013; Liu et al., 2018; Xu et al., 2020). In general, the membrane poration behavior is crucial for Mel to kill bacteria, which is a result of the complicated interplay between peptides and lipids.

The compositions of varying cell membrane systems are different. Specifically, cholesterol (Chol), which is almost absent in the bacterial membranes, is a key component of the animal cell membranes although changing in amount from <5 mol% to >40 mol% for different types of cells (Mouritsen and Zuckermann, 2004; van Meer et al., 2008). Chol plays crucial roles in stabilizing the membrane structure via maintaining the lipid ordering and membrane fluidity properties, etc. Chol is also associated with many fundamental cellular activities including signal transduction and intracellular trafficking. Containing Chol molecules or not is regarded as an important difference between mammalian and bacterial cells (Razin, 1975), which thus provides a potential target for AMPs’ selective attack for different cells. Unfortunately, it is still far from a better understanding of the AMPs’ action mechanism with a Chol-containing membrane or the molecular interactions between AMPs and Chols in a membrane environment.

In this work, the effects of Chol on Mel-membrane interactions are investigated by combining the dynamic giant unilamellar vesicle (GUV) leakage assay and coarse-grained (CG) molecular dynamics (MD) simulations. The inhibitory effect of Chol on the membrane poration behavior of Mel is observed in both experiments and simulations. Moreover, it is revealed by the simulations that Chols prefer to aggregate near the peptides and form peptide-Chol complexes in membrane. Under Mel actions, the location and orientation distributions of Chols in the two leaflets are asymmetrically perturbed. Furthermore, such changes in packing states of Chols are further dependent on the membrane environments or lipid compositions (e.g., saturated or unsaturated lipids), and the inhibitory effect of Chol is also changed correspondingly. Our results provide a molecular-level understanding of the Chol-AMP interaction mechanism and shed light on developing AMP-based membrane-targeting antibacterial agents for biomedical uses.

## Experimental Section

### Materials

1,2-dioleoyl-*sn*-glycero-3-phosphocholine (DOPC) and 1,2-dipalmitoyl-sn-glycero-3-phosphoethanolamine-N-(lissamine rhodamine B sulfonyl) (RhB-PE) were purchased from Avanti Polar Lipids. Mel, Chol and calcein were purchased from Sigma-Aldrich. All chemicals were used as received.

### Giant Unilamellar Vesicle Leakage Assay

GUVs, composed of pure DOPC or DOPC/Chol (at 7:3 by ratio), were prepared following the conventional electroformation method (Lu et al., 2019b; Xiao et al., 2019), washed with centrifugation (8000 rpm × 20 min, for three times), and diluted to a final concentration of ≈ 0.02 mg lipid per mL for use. The GUVs were then moved to a home-made cell, immobilized on the substrate surface for an in situ observation under confocal microscopy (LSM 710, Zeiss). Certain amount of saturated calcein solution was added to the GUV dispersion. After that, a fixed volume of peptide solution was injected gently, and the consequent calcein leakage was monitored in real time, in the lipid (EX 633 nm, EM 635–750 nm) and calcein (EX 488 nm, EM BP 530/50 nm) channels. All images were captured under the same instrumental settings. All the experiments were carried out at room temperature of 22°C (more details, refer to the Electronic Supplementary Information).

### Simulation Models

For Mel, its crystal structure was taken from the protein data bank (PDB ID: 2MLT). Specifically, chain A, which has a more flexible structure and consequently an improved membrane activity, was chosen (Irudayam and Berkowitz, 2012; Lyu et al., 2017; Deng et al., 2019; Xu et al., 2020). Then the peptide was coarse-grained using the Martinize script with MARTINI force field (version 2.2) (de Jong et al., 2013; Deng et al., 2019; Marrink et al., 2007). Furthermore, to realize the membrane poration in an accessible timescale, a bilayer consisting of the saturated CG MARTINI DLPC molecules or the unsaturated CG MARTINI DYPC molecules with a constant lipid number of 512 was constructed. Note that DLPC and DYPC have similar head groups and tail lengths. The only difference between them is that DLPC has saturated tails while DYPC has unsaturated ones instead. Thus, the comparison between them is helpful to examine the influence of lipid environment on the membrane poration behavior of Mel (Li et al., 2020). In addition, 0.1 M NaCl was included in the system and additional Cl^−^ ions were added to neutralize the charges of the peptides.

### Simulation Methods and Protocol

All simulations were performed by using the GROMACS 5.1.4 package with MARTINI force field (Hess et al., 2008). The semi-isotropic ensemble at a temperature of 310 K and a pressure of 1.0 bar were applied in the simulations. Specially, the temperature was controlled with the Berendsen temperature coupling scheme with a time constant of $\tau_{T}=1.0\;ps$ (Bussi et al., 2007), while the pressure was controlled using a Parrinello-Rahman semi-isotropic barostat with a time constant of 12 ps and a compressibility of 3 × 10^−4^ ${\text{bar}}^{-1}$ (Parrinello and Rahman, 1981). Periodic boundary conditions were applied in all three directions.

For all the simulation systems, the P/L ratios were kept to be 1/43 (i.e., 12 Mels and 512 lipids), which is exactly the threshold ratio for Mel to achieve membrane poration. The box size was set as ∼12.5 nm * 12.5 nm * 12.5 nm. At the beginning, the peptides were evenly placed above the bilayer surface (∼0.6 nm). Then the system was energy-minimized and equilibrated in the isothermal–isobaric (NPT) ensemble for 150 ns, with the location of peptides being fixed. All constraints were removed in the production runs and simulations were carried out for at least 10 μs with a time step of 20 fs.

Free energy was evaluated by reweighing the distribution of collective variables (CVs) with well-tempered metadynamics simulations, which were performed by using GROMACS package with PLUMED 2.2 plugin (Bonomi et al., 2009; Deng et al., 2019; Lelimousin et al., 2016; Tribello et al., 2014). Here, to describe the insertion situation of a peptide into a membrane, z-direction component of the distance (denoted as ΔZ) between center of mass (COM) of the peptide’s N-terminus and center of the bilayer was chosen as a CV.

### Statistical Analysis

All simulation and experimental measurements were performed with at least three to five replicates for each condition/data point. Analysis of variance was performed using OriginPro 9.0 (OriginLab software, Northampton, MA) and values are displayed as mean ± standard deviation.

## Results And Discussion

### Permeabilization of the Chol-Involved Membrane by Mel

**FIGURE 1:**
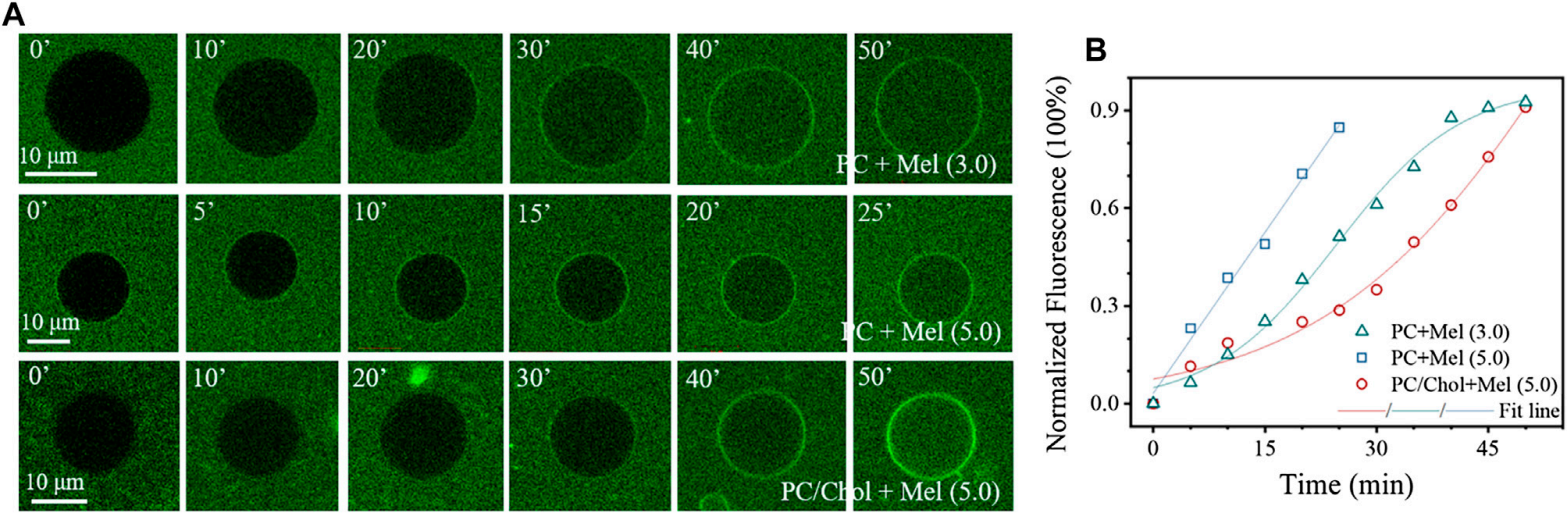
Permeabilization of GUV membranes by Mel. **(A)** Representative confocal images showing the time dynamic transmembrane entry process of calcein into GUVs composed of pure DOPC or DOPC/Chol (7/3 by ratio), upon Mel exposure at different concentrations. **(B)** Typical time-dependent intensity profiles showing increase of the normalized fluorescence intensity in the interior of GUVs upon Mel exposure. A sigmoidal or linear function is used to fit the profiles. Concentrations of the peptides are marked in the image (in µg mL^−1^). Times in **(A)** are in min.

The influence of Chol on Mel-membrane interactions was firstly investigated by the dynamic GUV leakage assay. Mel-induced transmembrane leakage of calcein occurs to GUVs composed of pure DOPC lipids once above a threshold peptide concentration of ∼3.0 µg mL^−1^. However, this concentration is increased to at least 5.0 µg mL^−1^ for the GUVs composed of DOPC and Chol. Figure 1A shows representative images during the dynamic transmembrane diffusion process of fluorescent probes (i.e., calcein) from the outside to the inside of a GUV, without or with Chol, upon peptide exposure. The corresponding time dependent distribution of the normalized fluorescence intensity of the interior of GUV (as an I∼t profile) in each case is representatively shown in Figure 1B, from which we can obviously see the difference in membrane permeabilization effect between cases. For the pure DOPC GUVs, an increased peptide concentration, e.g., from 3.0 to 5.0 µg mL^−1^, obviously enhances the membrane permeabilization effect, from a sigmoidal (i.e., $\text{I}=a/(1+e^{-k*(t-t_{c})}$)) to a linear (I=a+b*t) distribution of the I∼t profile. Here in the 3.0 µg mL^−1^ condition, the initial rising stage in I (i.e., t = 0∼15 min in Figure 1B) before linear increase (t = 15∼35 min) probably refers to a gradual peptide-accumulation process on membrane before stable transmembrane entry of dyes, which is normally observed in low-peptide-concentration conditions.^7,11^ In contrast, although at the same Mel concentration (e.g. 5.0 µg mL^−1^), the peptide-induced calcein leakage through a DOPC/Chol membrane is much slower than that through a pure DOPC one. Here, a sigmoidal function is also used to fit the I∼t profile as that in the low-concentrated (i.e., 3.0 µg mL^−1^) pure DOPC case. Moreover, in this condition, a much larger $t_{c}$ value (i.e., 58.6 min compared with 24.6 min in the 3.0 µg mL^−1^ DOPC case) is obtained indicating a prolonged peptide accumulation process before stable transmembrane entry of calcein (shown as the delayed linear-increase range). These results fully show the significant inhibition effect of Chol on the membrane permeabiliztion efficiency of Mel, which would be beneficial for the specific activity of Mel on bacterial rather than mammalian cell membranes.

### Inhibitory Effect of Chol on Membrane Poration Ability of Mel

**FIGURE 2:**
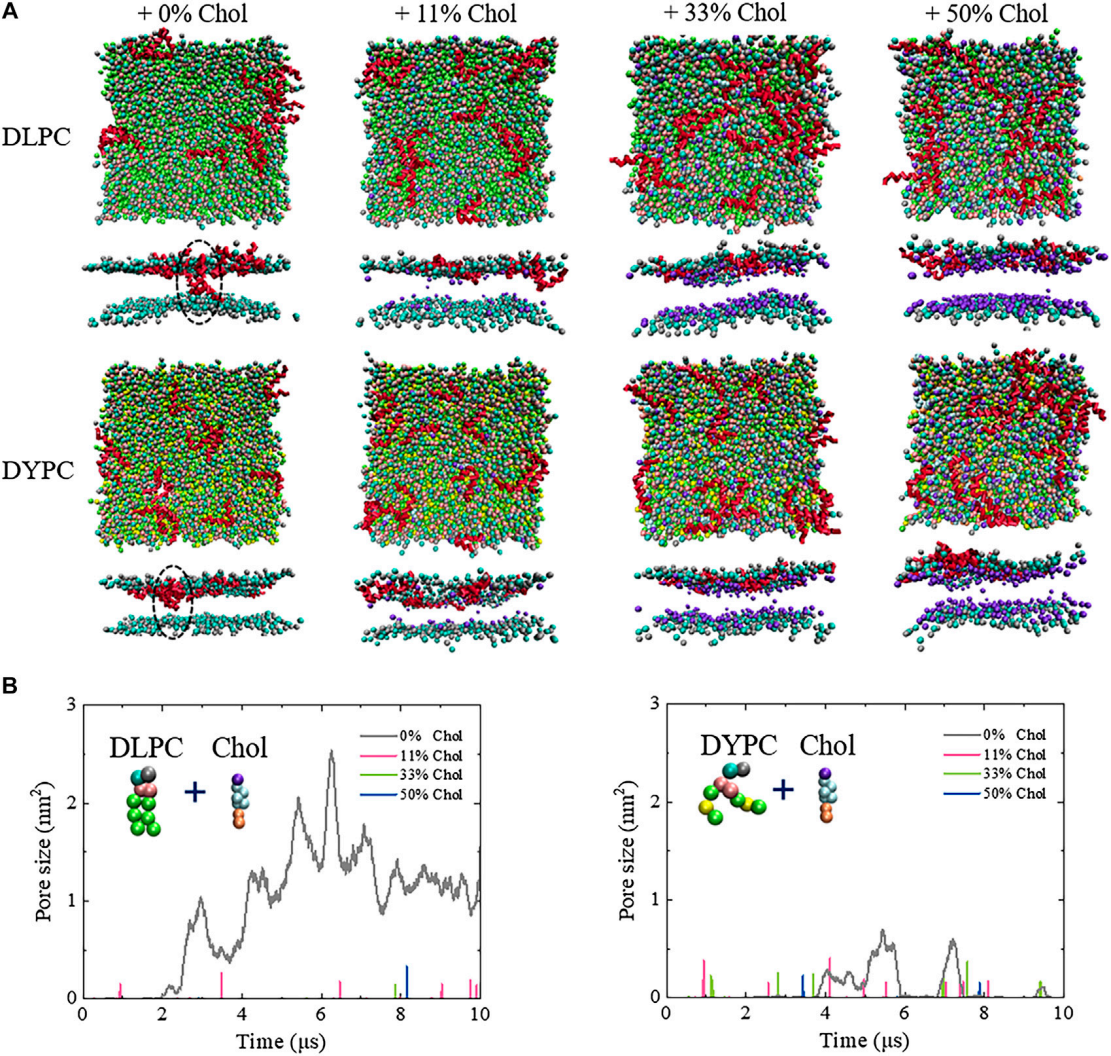
Inhibitory effect of Chol on the Mel-induced membrane poration. **(A)** Snapshots showing the interactions between Mel and a Chol-containing DLPC (top) or DYPC (bottom) membrane. Red: Mel. In each condition, the snapshot is shown in a top or side view. Lipid tails are not shown for clarity. All snapshots are obtained at ∼10 μs. Dashed circle highlights the membrane pore. **(B)** Evolution of pore size under the action of Mel. Left: DLPC membrane; Right: DYPC membrane. Insert shows lipid and Chol. The color codes of lipids and Chol are the same as **(A)**.

MD simulations, which have been proved powerful to gain in-depth insights into the molecular details in a membrane interaction process (Ding et al., 2012; Ding et al., 2018; Ji et al., 2016; Lu et al., 2019a), were performed to examine the interaction mechanism between Mel and Chol-containing membranes. As shown in Figure 2 and Supplementary Figures S1,S2, Mel indeed demonstrates totally different action modes with the membranes without or with Chol. For the pure membrane system, e.g., a DLPC lipid bilayer, Mel is able to make an obvious membrane pore at P/L=1/43. However, once Chols are included in the membrane system, the peptides only shallowly insert into the membrane and no membrane pore is observed in the same simulation conditions. Furthermore, the poration behavior of Mel is dependent on the lipid membrane environment. For the lipid bilayer consisting of unsaturated DYPC lipids, an unstable membrane pore with a smaller size is observed. The addition of Chol also hinders the pore formation induced by Mel, although the shallow insertion of peptides into this unsaturated lipid bilayer occurs more frequently.

**FIGURE 3:**
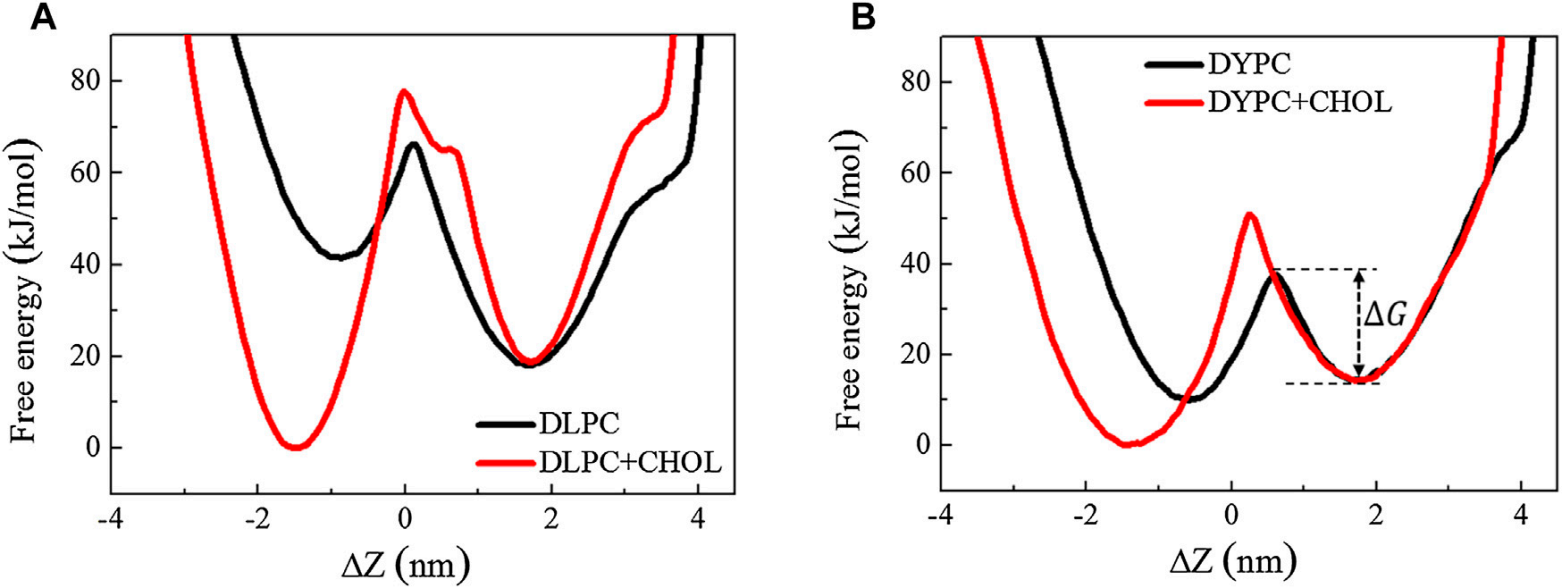
Free energy distribution of a Mel peptide during inserting into a lipid bilayer without or with Chol. The two minima correspond to the membrane-binding and -inserting states of the peptide, respectively. ΔG refers to the barrier for transition between these two states.

The inhibitory effect of Chol on the membrane poration activity of Mel is also reflected by the changes of free energy barrier, ΔG, during insertion of one Mel molecule into the membrane (Figure 3). As shown in Figure 3, the presence of Chol in the lipid bilayer increases the value of ΔG, indicating a larger difficulty for peptides to achieve membrane insertion. Moreover, for different membrane systems, the increasing degree of the barrier varies: for a saturated DLPC lipid bilayer, ΔG increases 23% due to Chol existence; while for an unsaturated DYPC lipid bilayer, such increase is approximately 58%. These results indicate that Chol has a stronger influence on the membrane translocation of Mel in a softer unsaturated lipid bilayer. That is, the Mel-Chol interactions are influenced by the membrane environment or lipid compositions.

### Mel-Lipid Interaction Influences the Chol Distribution in Membrane

**FIGURE 4:**
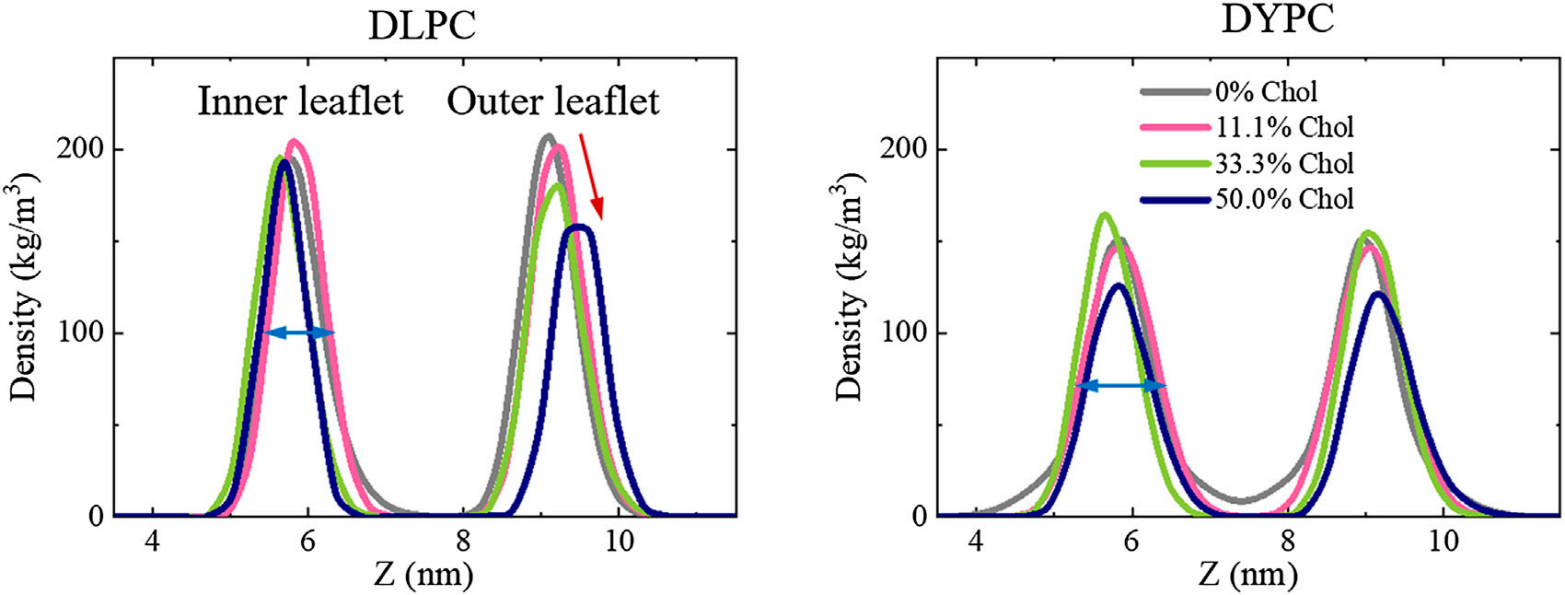
Peptide-induced perturbation on the bilayer structure of lipid membranes containing different amounts of Chol. Left: DLPC; Right: DYPC. For clarification, only the distributions of lipid heads (i.e., PO_4_ groups) are shown. Red and blue arrows show deviations in peak shape due to Chol contents or lipid species.

The poration behavior of Mel is tightly associated with the structural changes of membrane under peptide actions. Therefore, the possible influence of peptides on membrane structure was examined for a deep understanding of the inhibitory effect of Chol on peptide activity. As shown in Figure 4, for the pure DLPC lipid membrane, the bilayer structure is well kept even under the action of Mel. However, when Chol molecules are included in the membrane, the symmetry between two leaflets is broken under peptide actions. Specifically, the peak corresponding to the outer lipid leaflet, which directly interacts with peptides, becomes lower with the increase of Chol amount (marked with a red arrow in Figure 4). On the other hand, the DYPC membrane basically maintains the bilayer structure under peptide actions even when Chol is included in the system, although the peaks are lower and broader than that of a DLPC membrane (marked with blue arrows).

**FIGURE 5:**
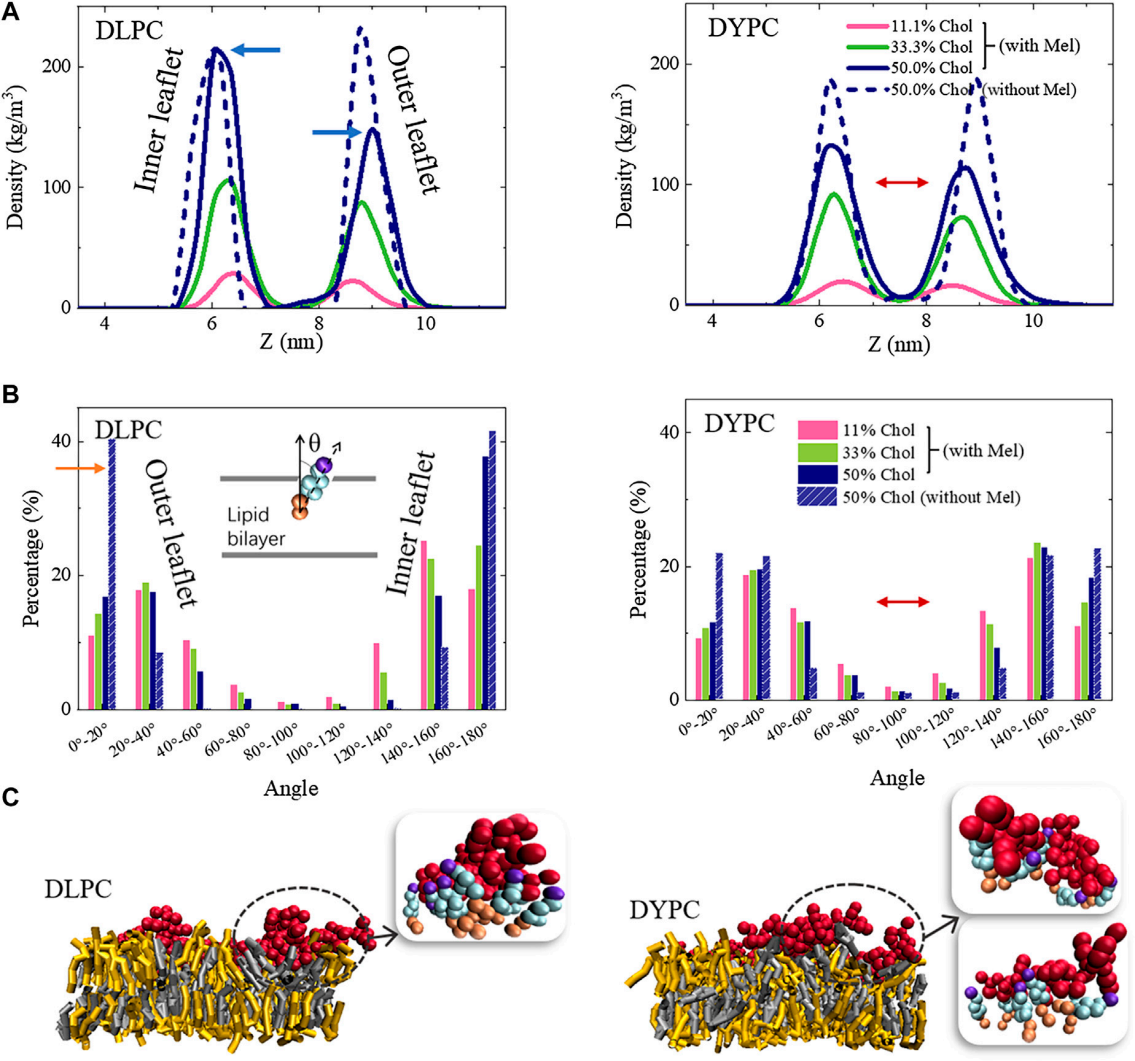
Packing state changes of Chol in membrane under Mel actions. **(A)** Location distribution of Chol in the two leaflets. **(B)** Orientation distribution of Chol. Left: DLPC; Right: DYPC. Inset in **(B)** shows the angle between Chol and normal direction of the bilayer. **(C)** Snapshots showing representative Mel-Chol interactions in different lipid membrane environments. Grey: DLPC; Yellow: Chol; Red: Mel. Insets (bead representation), Red: Mel; Purple, wathet and orange: head, ring and tail parts of Chol.

These observations suggest that, it is the Mel-Chol interaction that is mostly responsible for the changes of membrane structure. Thus, the distribution of Chol in membrane was further investigated. Note that in a pure DLPC bilayer without Mel, Chols distribute symmetrically in the two leaflets and keep vertical to the membrane plane (dashed lines in Figure 5A). However, under peptide actions, asymmetric distribution of Chol in the two leaflets occurs. It is found that the distribution peak of Chol in the outer leaflet becomes lower and broader, while that in the inner leaflets is barely affected (marked with blue arrows in Figure 5A). Meanwhile, Chols in the outer leaflet become obviously tilted (shown as the significant decrease of the 0-20° peak, marked with an orange arrow in Figure 5B) while the orientation changes in the inner leaflet is smaller. On the other hand, it is interestingly found that, for the DYPC lipid bilayer, the peptide action changes the distribution of Chol in both leaflets although they remain somewhat asymmetric (shown with red arrows in Figures 5A,B). In this condition, the Chol lipids are in a more mess state with diverging orientations compared with the DLPC case, which becomes even more chaotic upon Mel actions.

Molecular details of the interaction state between Mel and Chol molecules demonstrate that the distribution changes of Chol in bilayer are mainly caused by formation of Mel-Chol complexes in membrane. Obvious aggregation of Chol molecules around peptide chains is observed in both DLPC and DYPC membranes (Figure 5C). These Chol molecules normally attach the peptides closely and form a shell under the peptides, although the detailed shell structure differs in varying membrane environments. Therefore, the formation of such Mel-Chol complex is regarded as a main reason that blocks the further insertion of Mel into membrane and the pore formation. Besides this, by calculating order parameter of the acyl chains of lipids, it is found that DYPC and especially DLPC performs a much more compact packing state in membrane with Chol content increasing (Supplementary Figure S3), which would stiffen the bilayer structure and hinder the membrane insertion of molecules. Such effect might also make a contribution to the inhibitory effect of Chol on membrane poration ability of Mel.

## Conclusion

In this work, by combining experiments and MD simulations, the inhibitory effect of Chol on the membrane poration activity of Mel peptide, as well as the underlying molecular details, are investigated. The implication of Chol in lipid bilayer is demonstrated as an enhanced threshold working concentration of peptides, a decreased transmembrane leakage rate of dyes, and a larger free energy barrier for peptide insertion. Mel exposure might perturb the bilayer symmetry of the lipid membrane, and consequently change the tension status of the membrane. Such a structural perturbation is helpful for the membrane poration of Mels (Liu et al., 2018), which, however, might also stiffen the membrane and inhibit the peptide actions. Here, such an inhibitory impact is ascribed to the aggregation of Chol molecules in membrane around the membrane-bound peptide chains, and the consequent formation of peptide-Chol complexes which hinders the further insertion of Mel into bilayer. Our results provide an in-depth physical insight into the AMP-Chol interactions in the membrane environment, and suggest the possibility of developing advanced membrane-targeting antibacterial agents by making full use of the specific AMP-Chol interactions.

## Data Availability Statement

The original contributions presented in the study are included in the article/Supplementary Material further inquiries can be directed to the corresponding authors.

## Author Contributions

BY and KY conceived and designed the research. JL and WM performed the simulations and XL performed free energy calculation. JL, WM, BY, and KY analyzed the data. ZC, SS, and QW were responsible for collecting information. WM, BY, and KY wrote the paper.

## Funding

This work was financially supported by the National Natural Science Foundation of China (Nos. 21422404, 21774092, U1532108, U1932121, and 21728502) and the Priority Academic Program Development (PAPD) of Jiangsu Higher Education Institutions. BY and KY thank the support of the Natural Science Foundation of Jiangsu Province of China (Nos. BK20171207 and BK20171210).

## Supplementary Material

The Supplementary Material for this article can be found online at: https://www.frontiersin.org/articles/10.3389/fmolb.2021.638988/full#supplementary-material.

## Conflict of Interest

The authors declare that the research was conducted in the absence of any commercial or financial relationships that could be construed as a potential conflict of interest.

## References

[B1] Bonomi M. Branduardi D. Bussi G. Camilloni C. Provasi D. Raiteri P. (2009). PLUMED: A portable plugin for free-energy calculations with molecular dynamics. Comput. Phys. Commun 180, 1961–1972. 10.1016/j.cpc.2009.05.011

[B2] Bussi G. Donadio D. Parrinello M. (2007). Canonical sampling through velocity rescaling. J. Chem. Phys 126, 014101 10.1063/1.2408420 17212484

[B3] de Jong D. H. Singh G. Bennett W. F. D. Arnarez C. Wassenaar T. A. Schäfer L. V. (2013). Improved parameters for the martini coarse-grained protein force field. J. Chem. Theory Comput 9, 687–697. 10.1021/ct300646g 26589065

[B4] Deng Z. X. Li J. L. Yuan B. Yang K (2019). Residue-specialized membrane poration kinetics of melittin and its variants: insight from mechanistic landscapes. Commun. Theor. Phys. 71, 887–902. 10.1088/0253-6102/71/7/887

[B5] Ding H. Li J. Chen N. Hu X. Yang X. Guo L. (2018). DNA nanostructure-programmed like-charge attraction at the cell-membrane interface. ACS Cent. Sci. 4, 1344–1351. 10.1021/acscentsci.8b00383 30410972 PMC6202645

[B6] Ding H Tian W. Ma Y. (2012). Designing nanoparticle translocation through membranes by computer simulations. ACS Nano 6, 1230–1238. 10.1021/nn2038862 22208867

[B7] Duffy C. Sorolla A. Wang E. Golden E. Woodward E. Davern K. (2020). Honeybee venom and melittin suppress growth factor receptor activation in HER2-enriched and triple-negative breast cancer. Npj Precis. Oncol 4, 24 10.1038/s41698-020-00129-0 32923684 PMC7463160

[B8] Hess B. Kutzner C. van der Spoel D. Lindahl E. (2008). GROMACS 4: algorithms for highly efficient, load-balanced, and scalable molecular simulation. J. Chem. Theory Comput 4, 435–447. 10.1021/ct700301q 26620784

[B9] Hong J. Lu X. Deng Z. Xiao S. Yuan B. Yang K. (2019). How melittin inserts into cell membrane: conformational changes, inter-peptide cooperation, and disturbance on the membrane. Molecules 24, 1775 10.3390/molecules24091775 PMC6539814 31067828

[B10] Huang H. W. Chen F.-Y. Lee M.-T. (2004). Molecular mechanism of peptide-induced pores in membranes. Phys. Rev. Lett 92, 198304 10.1103/PhysRevLett.92.198304 15169456

[B11] Huang H. W. (2000). Action of antimicrobial peptides: two-state model^†^ . Biochemistry 39, 8347–8352. 10.1021/bi000946l 10913240

[B12] Irudayam S. J. Berkowitz M. L. (2012). Binding and reorientation of melittin in a POPC bilayer: computer simulations. Biochim. Biophys. Acta 1818, 2975–2981. 10.1016/j.bbamem.2012.07.026 22877705

[B13] Ji Q.-J. Yuan B. Lu X.-M. Yang K. Ma Y. Q. (2016). Controlling the nanoscale rotational behaviors of nanoparticles on the cell membranes: a computational model. Small 12, 1140–1146. 10.1002/smll.201501885 26436946

[B14] Krauson A. J. He J. Wimley W. C. (2012). Gain-of-function analogues of the pore-forming peptide melittin selected by orthogonal high-throughput screening. J. Am. Chem. Soc 134, 12732–12741. 10.1021/ja3042004 22731650 PMC3443472

[B15] Lam S. J. O’Brien-Simpson N. M. Pantarat N. Sulistio A. Wong E. H. H. Chen Y.-Y. (2016). Combating multidrug-resistant gram-negative bacteria with structurally nanoengineered antimicrobial peptide polymers. Nat. Microbiol 1, 16162 10.1038/nmicrobiol.2016.162 27617798

[B16] Lázár V. Martins A. Spohn R. Daruka L. Grézal G. Fekete G. (2018). Antibiotic-resistant bacteria show widespread collateral sensitivity to antimicrobial peptides. Nat. Microbiol 3, 718–731. 10.1038/s41564-018-0164-0 29795541 PMC6544545

[B17] Lee M. T. Sun T. L. Hung W. C. Huang H. W. (2013). Process of inducing pores in membranes by melittin. Proc. Natl. Acad. Sci. U.S.A 110, 14243–14248. 10.1073/pnas.1307010110 23940362 PMC3761581

[B18] Lelimousin M. Limongelli V. Sansom M. S. P. (2016). Conformational changes in the epidermal growth factor receptor: role of the transmembrane domain investigated by coarse-grained meta dynamics free energy calculations. J. Am. Chem. Soc 138, 10611–10622. 10.1021/jacs.6b05602 27459426 PMC5010359

[B19] Li W. Lin Z. Yuan B. Yang K. (2020). Tail-structure regulated phase behaviors of a lipid bilayer. Chinese Phys. B 29, 128701 10.1088/1674-1056/abad20

[B20] Liu J. Xiao S. Li J. Yuan B. Yang K. Ma Y. (2018). Molecular details on the intermediate states of melittin action on a cell membrane. Biochim. Biophys. Acta 1860, 2234–2241. 10.1016/j.bbamem.2018.09.007 30409519

[B21] Lu N. Yang K. Yuan B. Ma Y. (2012). Molecular response and cooperative behavior during the interactions of melittin with a membrane: dissipative quartz crystal microbalance experiments and simulations. J. Phys. Chem. B 116, 9432–9438. 10.1021/jp305141r 22794087

[B22] Lu X Xu P. Ding H. M. Yu Y. S. Huo D. Ma Y. Q. (2019a). Tailoring the component of protein corona via simple chemistry. Nat. Commun 10, 4520 10.1038/s41467-019-12470-5 31586045 PMC6778128

[B23] Lu X Liu J. Gou L. Li J. Yuan B. Yang K. (2019b). Designing melittin‐graphene hybrid complexes for enhanced antibacterial activity. Adv. Healthc. Mater 8, 1801521 10.1002/adhm.201801521 30866165

[B24] Lyu Y. Xiang N. Zhu X. Narsimhan G. (2017). Potential of mean force for insertion of antimicrobial peptide melittin into a pore in mixed DOPC/DOPG lipid bilayer by molecular dynamics simulation. J. Chem. Phys. 146, 155101 10.1063/1.4979613 28433027

[B25] Ma W. Sun S. Li W. Zhang Z. Lin Z. Xia Y. (2020). Individual roles of peptides PGLa and magainin 2 in synergistic membrane poration. Langmuir 36, 7190–7199. 10.1021/acs.langmuir.0c00194 32529830

[B26] Marrink S. J. Risselada H. J. Yefimov S. Tieleman D. P. de Vries A. H. (2007). The Martini force field: coarse grained model for biomolecular simulations. J. Phys. Chem. B 111, 7812–7824. 10.1021/jp071097f 17569554

[B27] Memariani H. Memariani M. Shahidi-Dadras M. Nasiri S. Akhavan M. M. Moravvej H. (2019). Melittin: from honeybees to superbugs. Appl. Microbiol. Biotechnol 103, 3265–3276. 10.1007/s00253-019-09698-y 30824944

[B28] Mouritsen O. G. Zuckermann M. J. (2004). What’s so special about cholesterol? Lipids 39, 1101–1113. 10.1007/s11745-004-1336-x 15726825

[B29] Munguia J. Nizet V. (2017). Pharmacological targeting of the host–pathogen interaction: alternatives to classical antibiotics to combat drug-resistant superbugs. Trends Pharmacol. Sci 38, 473–488. 10.1016/j.tips.2017.02.003 28283200 PMC5750058

[B30] Parrinello M. Rahman A. (1981). Polymorphic transitions in single crystals: a new molecular dynamics method. J. Appl. Phys 52, 7182–7190. 10.1063/1.328693

[B31] Ramadurai S. Holt A. Krasnikov V. van den Bogaart G. Killian J. A. Poolman B. (2009). Lateral diffusion of membrane proteins. J. Am. Chem. Soc 131, 12650–12656. 10.1021/ja902853g 19673517

[B32] Razin S. (1975). Cholesterol incorporation into bacterial membranes. J. Bacteriol 124, 570–572. 10.1128/JB.124.1.570-572.1975 809427 PMC235930

[B33] Sancho-Vaello E. Zeth K. (2015). Antimicrobial peptides: has their time arrived? Future Microbiol *.* 10, 1103–1106. 10.2217/fmb.15.45 26118303

[B34] Tribello G. A. Bonomi M. Branduardi D. Camilloni C. Bussi G. (2014). PLUMED 2: new feathers for an old bird. Comput. Phys. Commun 185, 604–613. 10.1016/j.cpc.2013.09.018

[B35] van Meer G. Voelker D. R. Feigenson G. W. (2008). Membrane lipids: Where they are and how they behave. Nat. Rev. Mol. Cell Biol 9, 112–124. 10.1038/nrm2330 18216768 PMC2642958

[B36] Willyard C. (2017). The drug-resistant bacteria that pose the greatest health threats. Nature 543, 15 10.1038/nature.2017.21550 28252092

[B37] Wimley W. C. (2010). Describing the mechanism of antimicrobial peptide action with the interfacial activity model. ACS Chem. Biol 5, 905–917. 10.1021/cb1001558 20698568 PMC2955829

[B38] Xiao S. Lu X. Gou L. Li J. Ma Y. Liu J. (2019). Graphene oxide as antibacterial sensitizer: mechanically disturbed cell membrane for enhanced poration efficiency of melittin. Carbon 149, 248–256. 10.1016/j.carbon.2019.04.067

[B39] Xu C. Ma W. Wang K. He K. Chen Z. Liu J. (2020). Correlation between single-molecule dynamics and biological functions of antimicrobial peptide melittin. J. Phys. Chem. Lett 11, 4834–4841. 10.1021/acs.jpclett.0c01169 32478521

[B40] Xu D. Jiang L. Singh A. Dustin D. Yang M. Liu L. (2015). Designed supramolecular filamentous peptides: balance of nanostructure, cytotoxicity and antimicrobial activity. Chem. Commun 51, 1289–1292. 10.1039/C4CC08808E PMC4866517 25476705

[B41] Yang L Harroun T. A. Weiss T. M. Ding L. Huang H. W. (2001). Barrel-stave model or toroidal model? a case study on melittin pores. Biophys. J 81, 1475–1485. 10.1016/S0006-3495(01)75802-X 11509361 PMC1301626

[B42] Zhang Y. Chen T. Pan Z. Sun X. Yin X. He M. (2018). Theoretical insights into the interactions between star-shaped antimicrobial polypeptides and bacterial membranes. Langmuir 34, 13438–13448. 10.1021/acs.langmuir.8b02677 30350688

