# Supplementary material for: Cholesterols Work as a Molecular Regulator of the Antimicrobial Peptide-Membrane Interactions
Source: Front Mol Biosci. 2021 Feb 3;8:638988. doi: 10.3389/fmolb.2021.638988 (PMC7902056; doi:10.3389/fmolb.2021.638988)
Supplement: Supplementary file 1 [file datasheet1.pdf]

SUPPLEMENTARY MATERIAL for

Cholesterols work as a molecular regulator of the antimicrobial peptide-membrane interactions

Jia Li[†,1], Xuemei Lu[†,2,3], Wendong Ma[†,3], Zhonglan Chen[3], Shuqing Sun[3], Qinghui Wang[1], Bing Yuan[*,3], and Kai Yang[*,3]

[1]Electric & Informative Engineering School, Yunnan Minzu University, Kunming, 650504, P. R. China

[2]Wenzheng College of Soochow University, Suzhou, 215006, P. R. China

[3]Center for Soft Condensed Matter Physics and Interdisciplinary Research & School of Physical Science and Technology, Soochow University, Suzhou, 215006, P. R. China

1 EXPERIMENTAL DETAILS

1.1 GUV leakage assay

Fabrication of GUVs. GUVs were prepared following the conventional electro-formation method. Briefly, a solution of lipids (DOPC containing 0.1 mol% RhB-PE for fluorescence labeling; 60 μL × 2.0 mg mL^{-1} in chloroform) was deposited onto two ITO-coated glass slides and dried under vacuum overnight. The dry film was transferred in a homemade electro-formation chamber (with the two glass slides as electrodes), and rehydrated in 0.1 M sucrose buffer. Alternating voltages were applied (0.5 V × 20 min, 1.0 V × 20 min and 1.5 V × 3 h). The obtained vesicles were washed three times via centrifugation (8000 rpm × 20 min). Well dispersed GUVs with a size distribution of 8−30 μm were collected (~0.02 mg lipid mL^{-1}) and transferred to a sample chamber for observation.

Immobilization of GUVs for in situ observation. A home-made chamber cell, with a cover-glass substrate, was used for microscope observation in the experiments. For immobilization of the vesicles, the glass substrate was pre-treated with the following procedures. First, the glass slide was washed completely with ethanol and water consequently, boiled in a mixture of H_2O_2 and H_2SO_4 (3:7 by vol) for 1 h, washed again with a large amount of water followed by drying with N_2 flow. It was then dipped into APTES for 5 min, and dried under N_2 flow again. After that, the glass slide was kept at 120 °C for 30 min, before being installed in the chamber for use. For the in-situ microscopy observations, a volume of GUV dispersion was pretransferred to the chamber cell and stabilized for about 5 min for particle immobilization. After that, certain amount of calcein and peptide solutions were added for the in situ monitoring under confocal microscopy.

Data collection and analysis. During the dynamic entry process of calcein into the interior of a GUV due to drug addition, fluorescence intensity of the GUV interior at each time point (i.e., mean value among pixels read out directly from the Zeiss LSM software) was normalized with that of the surrounding environment and plotted as a function of time.

2 SUPPLEMENTARY IMAGES

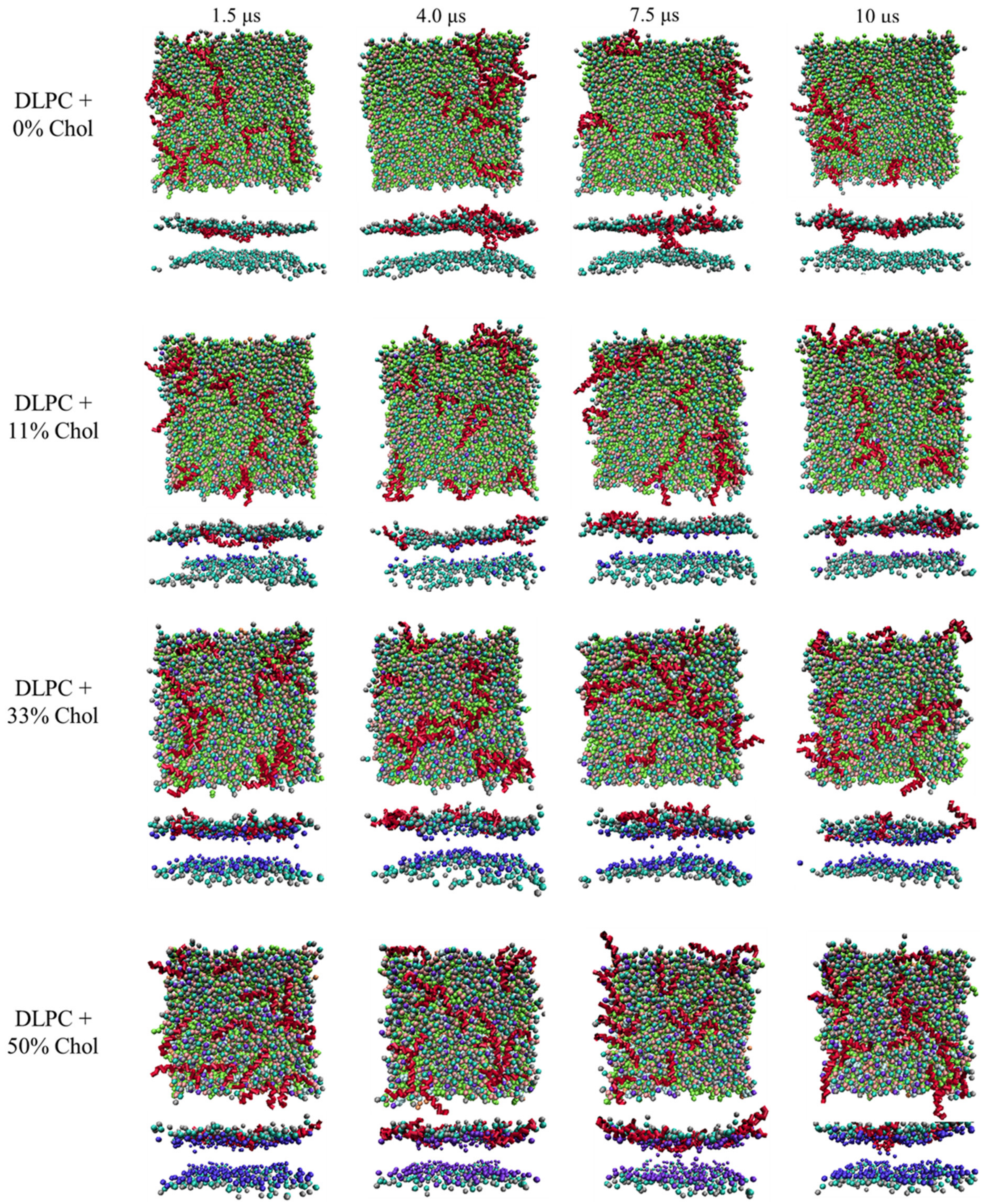

FIGURE S1 | Interactions between Mel and DLPC lipid bilayers with varying amount of Chols.

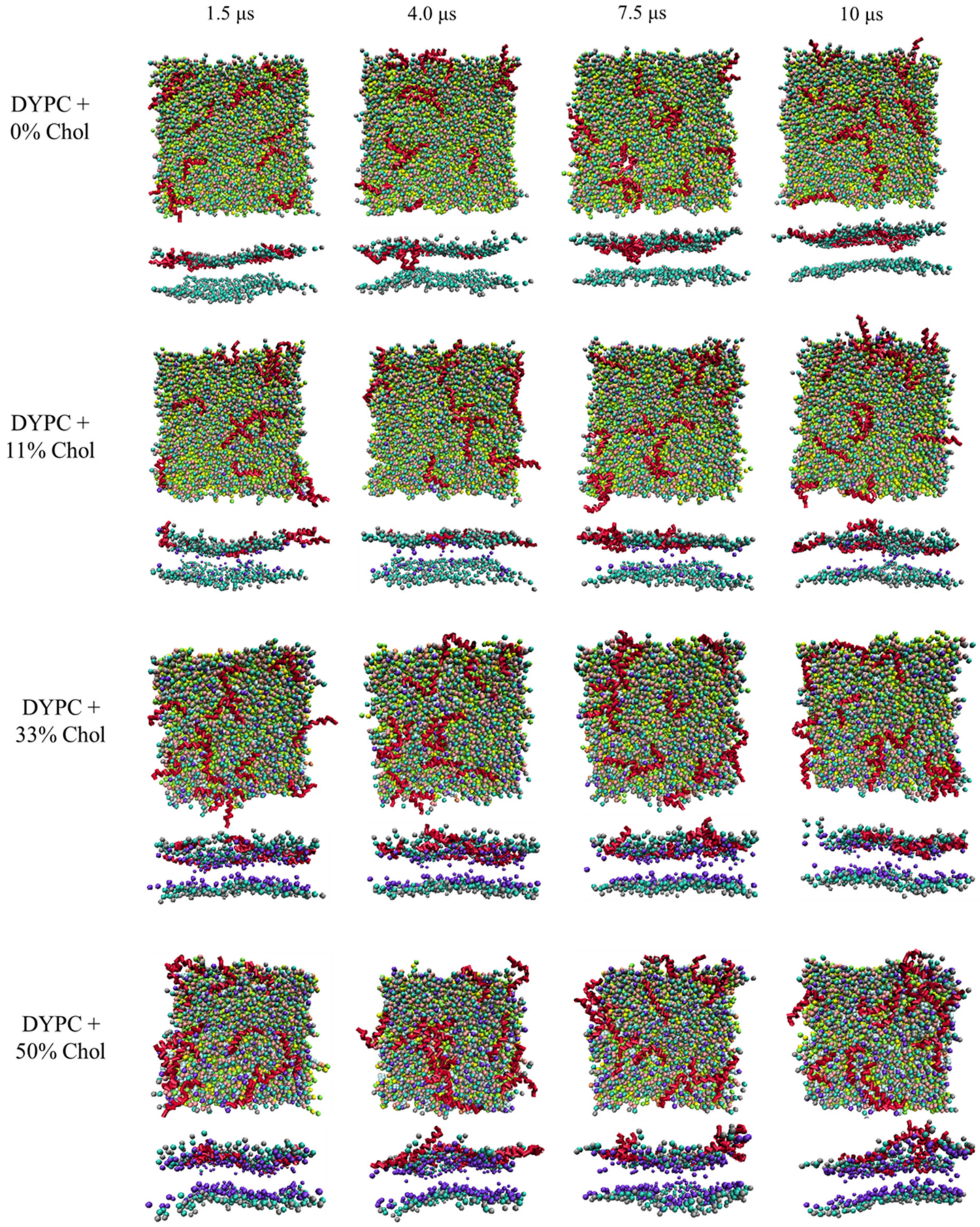

FIGURE S2 | Interactions between Mel and DYPC lipid bilayers with varying amount of Chols.

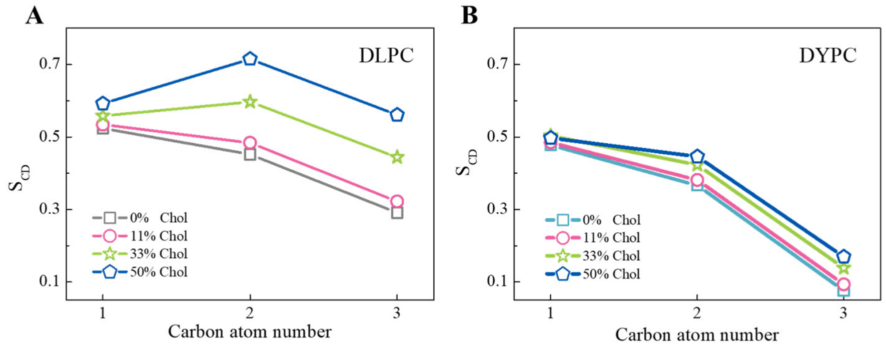

FIGURE S3 | Changes of order parameters (S_{CD}) of (A) DLPC and (B) DYPC under the varying Chol concentrations.
